# Genome Sequence of the Pseudomonas protegens Phage ΦGP100

**DOI:** 10.1128/genomeA.00261-18

**Published:** 2018-06-21

**Authors:** Jordan Vacheron, Peter Kupferschmied, Grégory Resch, Christoph Keel

**Affiliations:** a Department of Fundamental Microbiology, University of Lausanne, Lausanne, Switzerland

## Abstract

We report here the complete annotated genome sequence of ΦGP100, a lytic bacteriophage of the Podoviridae family. ΦGP100 was isolated from rhizosphere soil in Switzerland and infects specifically strains of Pseudomonas protegens that are known for their plant-beneficial activities. Phage ΦGP100 has a 50,547-bp genome with 76 predicted open reading frames.

## GENOME ANNOUNCEMENT

In 2002, Keel and colleagues isolated a lytic bacteriophage belonging to the Podoviridae family from the rhizosphere of cucumber plants (1). The phage, named ΦGP100, was found to infect specifically Pseudomonas protegens CHA0 and related strains of the same species. P. protegens strains are highly competitive root colonizers and are studied for their biocontrol effects against plant pathogens (2, 3, 4) and herbivorous insect pests (5, 6).

We sequenced and annotated the full genome of ΦGP100. Extraction of ΦGP100 DNA was done from a purified suspension of the phage containing 10^9^ PFU · ml^−1^ using a standard phenol-chloroform extraction procedure. The phage DNA was sequenced at the Lausanne Genomic Technologies Facility in Switzerland. Sequencing libraries were prepared using the TruSeq Nano DNA LT library preparation kit (Illumina, San Diego, CA, USA) and sequenced with the HiSeq 2500 platform, generating an output of 100-bp paired-end reads. Reads were assembled into contigs with the Edena v3 *de novo* short read assembler (7). Annotation of open reading frames (ORFs) was done with Rapid Annotations using Subsystems Technology (RAST) (8) and PHAge Search Tool Enhanced Release (PHASTER) (9). Each predicted ORF was further examined using BLAST and Conserved Domain database searches on the NCBI website (https://www.ncbi.nlm.nih.gov). tRNAs were predicted using ARAGORN (10).

A total of 20,139,130 paired-end reads were obtained, leading to a coverage exceeding 39,500×. The assembly generated a single contig of 50,547 bp with a G+C content of 51% corresponding to the entire phage genome, which is in agreement with the genome size previously determined by restriction analysis (1). Seventy-six potential ORFs were predicted. In particular, we found structural genes coding for phage tail fiber protein (GenBank accession number SPF82154), phage terminase large subunit (SPF82151), phage portal protein (SPF82150), and phage capsid protein (SPF82132). We also found genes encoding proteins potentially involved in phage DNA replication, notably a DNA helicase (GenBank accession number SPP13286), a polymerase (SPF82110), and a lysin for phage release (SFP82136). Two tRNA sequences were predicted, one of which is a 73-nucleotide (nt)-long tRNA for which the anticodon reads a stop codon (TAA), suggesting that it may act as a nonsense suppressor (11). The anticodon of the second predicted tRNA reads an Asn codon (GTT).

The best nucleotide BLAST hits for the whole genome were Pseudomonas phage IME180 (GenBank accession number MF788075) and Pseudomonas phage O4 (NC_031274), which shared less than 70% identity on maximally 32% of their genome lengths with the ΦGP100 genome. All of these phages infect P. aeruginosa strains, unlike phage ΦGP100, which seems to be specific to a subset of *P. protegens* strains (1; our unpublished data). Phages can be considered a major driving force influencing microbial diversity in soil (12), and the ecological study of this phage-Pseudomonas model may thus lead, in a larger perspective, to an improved understanding of phage-bacterium interactions in complex environments such as the rhizosphere.

### Accession number(s).

The complete genome sequence of ΦGP100 was deposited at the European Nucleotide Archive as BioProject ID PRJEB24648, sample ERS2161702. The assembled genome sequence was deposited at DDBJ/EMBL/GenBank under the accession number LT986460.
